# Target-site *EPSPS* Pro-106-Ser mutation in *Conyza canadensis* biotypes with extreme resistance to glyphosate in Ohio and Iowa, USA

**DOI:** 10.1038/s41598-020-64458-7

**Published:** 2020-05-05

**Authors:** Zachery T. Beres, Laura A. Giese, David M. Mackey, Micheal D. K. Owen, Eric R. Page, Allison A. Snow

**Affiliations:** 1 Department of Evolution, Ecology, and Organismal Biology; Ohio State University, Columbus, OH 43210 USA; 2 Department of Horticulture and Crop Science; Ohio State University, Columbus, OH 43210 USA; 3 Department of Agronomy; Iowa State University, Ames, IA 50011 USA; 4 Harrow Research and Devleopment Centre, Agriculture and Agri-Food Canada, Harrow, ON N8H 4W7 Canada

**Keywords:** Agroecology, Plant ecology, Plant evolution

## Abstract

Documenting the diversity of mechanisms for herbicide resistance in agricultural weeds is helpful for understanding evolutionary processes that contribute to weed management problems. More than 40 species have evolved resistance to glyphosate, and at least 13 species have a target-site mutation at position 106 of *EPSPS*. In horseweed (*Conyza canadensis*), this p106 mutation has only been reported in Canada. Here, we sampled seeds from one plant (= biotype) at 24 sites in Ohio and 20 in Iowa, screened these biotypes for levels of resistance, and sequenced their DNA to detect the p106 mutation. Resistance categories were based on 80% survival at five glyphosate doses: S (0×), R1 (1×), R2 (8×), R3 (20×), or R4 (40×). The p106 mutation was not found in the19 biotypes scored as S, R1, or R2, while all 25 biotypes scored as R3 or R4 had the same proline-to-serine substitution at p106. These findings represent the first documented case of target-site mediated glyphosate resistance in horseweed in the United States, and the first to show that this mutation was associated with very strong resistance. We hypothesize that the p106 mutation has occurred multiple times in horseweed and may be spreading rapidly, further complicating weed management efforts.

## Introduction

Widespread application of herbicides has spurred the rapid and repeated evolution of herbicide resistance in agricultural weeds, and at least 43 weed species have evolved glyphosate resistance^1^. Arguably, no herbicide has had a larger impact on agricultural weed management practices in the last few decades or been more widely applied than glyphosate, the active ingredient in RoundUp^2,3^. As a non-selective, post-emergent herbicide, glyphosate inhibits 5-enolpyruvylshikimate-3-phosphate synthase (EPSPS), a key enzyme in the shikimate acid pathway required for aromatic amino acid synthesis^2^. The majority of all maize, soybean, cotton, sugar beet, and canola planted in the United States is glyphosate resistant^3^. The total amount of glyphosate applied in the United States has risen drastically over the past two decades, and the amount of glyphosate applied per hectare in soybean fields more than doubled from 1995 to 2015^3^.

**Figure 1:**
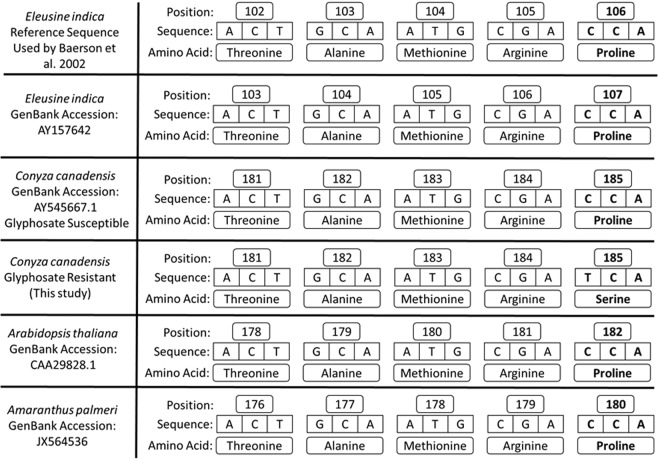
Conserved *EPSPS*2 sequences of *Eleusine indica, Conyza canadensis, Arabidopsis thaliana*, and *Amaranthus palmeri*. Each species is reported as having the Pro-106-Ser point mutation in cited publications, although position numbers from GenBank are different, as shown here. The highly conserved (homologous) region of amino acids with their respective positions are shown for each species. *Elusine indica* is included as the original “reference sequence” because Baerson *et al*.^6^ designated the mutation as p106, although this was based on a microbial sequence (see text). A glyphosate-resistant biotype of *C. canadensis* is included to illustrate the proline-to-serine mutation that confers glyphosate resistance.

Agricultural weeds have evolved several mechanisms of resistance to glyphosate, which are generally grouped into two distinct categories: non-target site and target-site^4,5^. Non-target site mechanisms involve metabolism, altered translocation, and vacuolar sequestration, while target-site mechanisms generally include amplification of *EPSPS*, overexpression of *EPSPS*, and amino acid substitutions in *EPSPS*^4,5^. Point mutations conferring glyphosate resistance have been well-documented in several weed species; the most common substitution is a change of a conserved proline at position 106 (p106) of *EPSPS* to one of four other amino acids (serine, threonine, alanine, or leucine^4,5^; Table 1). This mutation was first reported in *Eleusine indica* (goosegrass) by Baerson *et al*.^6^, who noted that it lies within a highly conserved region of amino acids found within all plants’ and most bacterial *EPSPS* genes. For *E. indica*, Baerson *et al*.^6^ aligned the sequence of amino acids in this region with the numbering system for *Escherichia coli* and other microbes, rather than the true sequence for *E. indica* (Fig. 1), and referred to it as p106. This labelling practice has largely been adopted by the scientific community and is used here, as we discuss in the Results and Discussion.

**Table 1:** Examples of weed species with target-site resistance to glyphosate endowed by a point mutation.

Species	Reference	State, Country	Pro106^a^ to	Additional mutations?
*Amaranthus hybridus*	^12^	Argentina	Ser	Thr-102-Ile, Ala-103-Val^b^
*Amaranthus palmeri*	^46^	Mexico	Ser	
*Amaranthus tuberculatus*	^47^	Illinois, USA	Ser	
	^33^	Mississippi, USA	Ser	
	^48^	Ohio, USA	Ser	
*Bidens pilosa*	^7^	Puebla, Mexico	Ser	Thr-102-Ile^cd^
*Chloris virgata*	^49^	Australia	Ser	Pro-106-Leu
*Conyza canadensis*	^23^	Canada	Ser	
*Digitaria insularis*	^50^	Brazil	Thr^e^	
*Echinochloa colona*	^51^	California, USA	Ser	
	^52^	California, USA	Ser	Pro-106-Thr
*Eleusine indica*	^6^	Malaysia	Ser	
	^53^	Malaysia	Ser	Pro-106-Thr
	^54^	Malaysia	Ser	Pro-106-Thr
	^55^	Philippines	Ser	
	^8^	Jerantut, Malaysia	Ser	Thr-102-Ile^c^
	^56^	Veracruz, Mexico	Ser	
	^9^	Australia	Ser	Thr-102-Ile^cd^
	^10^	China	Ser	Thr-102-Ile; Pro-106-Leu^cf^
	^11^	China	Ser	Thr-102-Ile; Pro-106-Leu^cf^
	^57^	Brazil	Ser	
*Leptochloa virgata*	^58^	Veracruz, Mexico	Ser	
*Lolium multiflorum*	^59^	Chile	Ser	
	^60^	California, USA	Ser	Pro-106-Ala
	^39^	Spain	Ser^e^	
*Lolium rigidum*	^61^	Australia	Thr	
	^62^	South Africa	Ala	
	^63^	California, USA	Ser^e^	
	^35^	South Africa	Leu	
	^34^	Australia	Ser	Pro-106-Thr
	^64^	Italy	Ser	Pro-106-Leu; Leu-106-Ser
*Poa annua*	^65^	South Carolina, USA	Ala	

Note: Species with multiple mutations reported at p106 occur within separate individuals.

^a^Numbering scheme established in Baerson *et al*.^6^.

^b^TAP-IVS (Thr-102-Ile, Ala-103-Val, Pro-106-Ser) mutation occurs in individual plants.

^c^TIPS (Thr-102-Ile, Pro-106-Ser) mutation occurs in individual plants.

^d^Pro-106-Ser mutation also identified in individual plants.

^e^Mutation not reported at p106 but is equivalent; see Results and Discussion.

^f^Pro-106-Leu mutation identified in individual plants.

In addition to glyphosate resistance mutations at p106, there are reports of additional point mutations occurring at p102 and p103. For example, a double point mutation (threonine to isoleucine at p102 and proline to serine at p106) was found in individuals of *Bidens pilosa* L. from Mexico and *Eleusine indica* from Malaysia, Australia, and China^7–11^ (Table 1). This double point mutation, often referred to as the TIPS mutation, is associated with increased glyphosate resistance^4,5^. Recently, a triple point mutation (threonine to isoleucine at p102, alanine to valine at p103, and proline to serine at p106) was found as the sole glyphosate resistance mechanism within individuals of *Amaranthus hybridus* L. from Argentina^12^.

*Conyza canadensis*, known as horseweed or marestail, was the first broadleaf weed to evolve glyphosate resistance, first observed in 2000 in no-tillage soybean fields in Delaware, USA^13^. Since then, glyphosate resistance in horseweed has been documented in 25 states within the USA and 12 countries worldwide^1^. As one of the first glyphosate-resistant weeds identified, *C. canadensis* has been the focus of many studies on the specific mechanisms of glyphosate resistance. Early reports indicated that glyphosate resistance was controlled by an incompletely dominant, single-locus nuclear allele^14,15^. Initial reports, which were later confirmed by other studies, determined that horseweed was able to absorb glyphosate and rapidly sequester it within the vacuoles of its leaves, and this was determined to be “the horseweed resistance mechanism”^16,17^. Indeed, non-target site resistance mechanisms (generally altered translocation and vacuolar sequestration) have been found in horseweed samples from Arkansas, California, Delaware, Iowa, Mississippi, Ohio, Virginia, and Washington in the USA, as well as in Canada, Spain, and Greece^16–27^. Several studies have linked vacuolar sequestration to an active process involving transporter genes that are induced by glyphosate treatment^24,26,28^.

However, there also have been scattered reports of other non-target and target-site mechanisms in *C. canadensis*. A study by Ge *et al*.^29^ reported an unknown resistance mechanism in a biotype from Delaware conferring low-levels of resistance. González-Torralva *et al*.^22^ documented horseweed biotypes in Spain that metabolized glyphosate. In addition to having reduced translocation, horseweed biotypes from Ohio, Delaware, Arkansas, and Virginia were found to have 2–3-fold greater levels of basal *EPSPS* mRNA in glyphosate resistant biotypes^19^. Similarly, Tani *et al*.^28^ reported 2–3-fold greater *EPSPS* expression in a resistant biotype from Greece. However, another study from Greece^30^ did not find increased *EPSPS* transcript levels in two resistant biotypes, and Peng *et al*.^25^ did not detect multiple copies of *EPSPS* in a resistant biotype from Tennessee, USA.

Horseweed has three *EPSPS* genes [i.e., *EPSPS*1 (AY545666.1), *EPSPS*2 (AY545667.1), and *EPSPS*3 (AY545668.1)^23^], and several studies have attempted to identify a point mutation conferring glyphosate resistance in horseweed. The first target-site mediated glyphosate resistance in horseweed was reported in Canada in 2018 by Page *et al*.^23^. Page and colleagues identified the proline to serine mutation at p106 of *EPSPS*2 in 21 glyphosate resistant populations sequenced at *EPSPS*2. However, other researchers did not detect this point mutation in horseweed populations sampled from Crete and Lakonia in Greece, from Delaware in the United States, or from the Beijing and Shandong provinces in China^28,30,31^.

Many of the studies cited above examined relatively few biotypes and did not account for the broad range of glyphosate resistance levels found within horseweed. We had previously screened single-plant seed accessions (=biotypes) of *Conyza canadensis* across northcentral Ohio and southern Iowa. In these screenings, we found that glyphosate resistance to up to 40x the manufacturer’s recommended application rate (1x= 840 g ae ha^−1^) was quite common in both states^32^. These very high levels of glyphosate resistance, along with the recent finding of a point mutation in Canada, prompted us to sequence our horseweed biotypes from Ohio and Iowa to search for a similar point mutation. Unlike previous studies investigating point mutations in horseweed, including Page *et al*.^23^, we characterized different levels of glyphosate resistance based on 80% survival at various dosages: 0x(S = Susceptible), 1x(R1), 8x(R2), 20x(R3), and 40x(R4), and we sequenced a large number of biotypes.

The main questions addressed by the study were:

1. Does a point mutation occur in horseweed biotypes originating from northcentral Ohio and southern Iowa?
2. If so, is the same point mutation (Pro-106-Ser) found in biotypes from Ohio, Iowa, and Canada?
3. Is the point mutation found in biotypes with all levels of glyphosate resistance (R1–R4)?

## Results and Discussion

The presence or absence of the proline to serine mutation at position 106 was associated with glyphosate resistance categories. None of the biotypes from Ohio and Iowa that were scored as susceptible (S), R1, or R2 had a point mutation at p106 of the *EPSPS*2 gene, while all biotypes from Ohio and Iowa that were scored as either R3 or R4 did have the proline to serine substitution at p106 (all were CCA to TCA; Table 2). Likewise, the presence or absence of the point mutation matched all Canadian accessions based on glyphosate resistance category, with the exception of one R4 accession (CA 27) which, upon screening an additional 6 individual plants was found to have a mixture of genotypes, some with the p106 mutation and others lacking it (Table 2).

**Table 2:** Samples of *Conyza canadensis* from Ohio, Iowa, and Canada exhibiting either proline or serine at position 106 of *EPSPS*2.

Region	Amino acid at p106	First base at p106	Sample ID	Resistance rank	Amino acid at p106	First base at p106	Sample ID	Resistance rank
**Ohio**	Proline	**C**	N64	**S**	Serine	**T**	N89	**R3**
		**C**	N28	**S**		**T**	N11	**R4**
		**C**	N50	**S**		**T**	S15	**R4**
		**C**	N53	**S**		**T**	S18	**R4**
		**C**	N58	**S**		**T**	S23	**R4**
		**C**	N66	**S**		**T**	N26	**R4**
		**C**	N90	**S**		**T**	S3	**R4**
		**C**	N42	**R1**		**T**	S38	**R4**
		**C**	N83	**R1**		**T**	S60	**R4**
		**C**	N52	**R2**		**T**	N65	**R4**
						**T**	S74	**R4**
						**T**	N76	**R4**
						**T**	S78	**R4**
						**T**	N9	**R4**
**Iowa**	Proline	**C**	N17	**S**	Serine	**T**	N10	**R3**
		**C**	N2	**S**		**T**	S12	**R3**
		**C**	N9	**S**		**T**	S9	**R3**
		**C**	N12	**R1**		**T**	N28	**R4**
		**C**	N19	**R1**		**T**	S11	**R4**
		**C**	S16	**R1**		**T**	S14	**R4**
		**C**	S4	**R1**		**T**	S15	**R4**
		**C**	S8	**R1**		**T**	S25	**R4**
		**C**	S37	**R2**		**T**	S27	**R4**
						**T**	S41	**R4**
						**T**	S45	**R4**
Canada^*^	Proline	**C**	59	**S**	Serine	**T**	20	**R3**
		**C**	67	**S**		**T**	10	**R4**
		**C**	75	**S**		**T**	13	**R4**
		**C**	79	**S**		**T**	15	**R4**
		**C**	80	**R1**^**1**^				

Biotypes or accessions were grouped into resistance categories based on <80% survival at 1×(S) or ≥80% survival at 1×(R1), 8×(R2), 20×(R3), and 40×(R4); see Beres *et al*.^32^ for details. “S” and “N” in sample IDs designate samples collected from “Soybean fields” or “Non-agricultural sites,” respectively. All R1 and R2 plants are presumed to have non-target site resistance, which could also occur in R3 and R4.

^1^Scored as susceptible by Page *et al*.^23^; this accession may represent multiple individuals and may have non-target resistance.

^*^One accession (CA 27; R4) is not shown because it respresented multiple individuals, some with the p106 mutation and others lacking it.

While we did not test the Ohio and Iowa biotypes for altered translocation and vacuolar sequestration, we presume that biotypes at all levels of resistance (R1–R4) would most likely possess one or more non-target site mechanisms of resistance. We base this assertion on the fact that every previous study which has looked for altered translocation and vacuolar sequestration within glyphosate resistant horseweed populations has found ite.g.^16–19,21,22,24–26^.

To our knowledge, these findings represent the first documentation of target-site mediated glyphosate resistance in horseweed within the United States. The fact that the Pro-106-Ser point mutation was only found in biotypes with the most extreme levels of resistance (R3 [≥80% survival at 20x] and R4 [≥ 80% survival at 40x]) is noteworthy. Individually, target-site mechanisms are typically considered to confer low levels of glyphosate resistance in other species^4^, although there have been reports of a p106 point mutation working synergistically with non-target or other unknown mechanisms to provide increased levels of glyphosate resistance^33–35^. In the present study, the Pro-106-Ser mutation was associated with extreme levels of glyphosate resistance (tolerating 20x−40x; Table 2). Further research could be carried out to ascertain whether such high levels of resistance require multiple resistance mechanisms or simply a mutation at p106.

**Figure 2:**
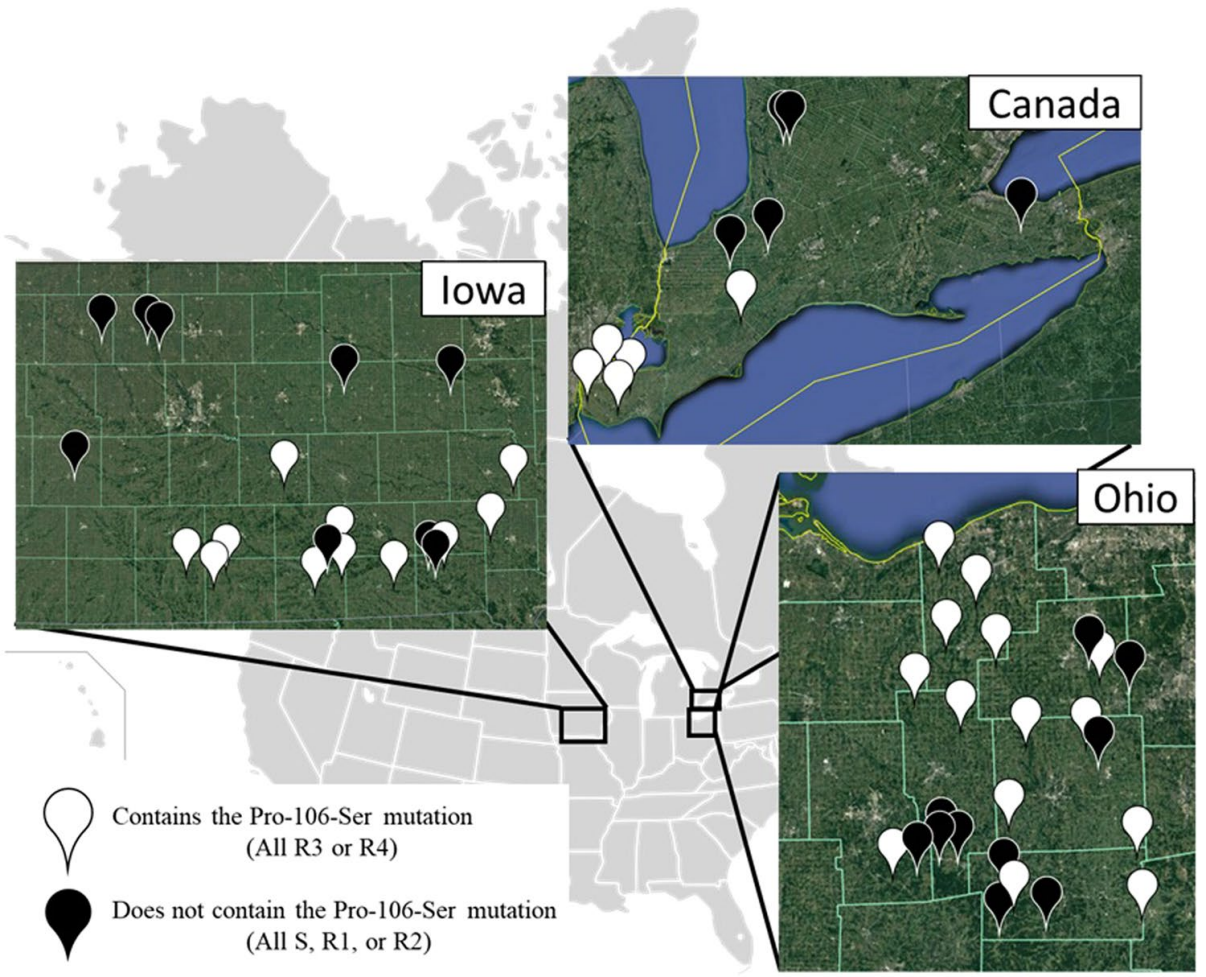
Map showing location of *Conyza* biotypes and accessions in this study and the presence or absence of the Pro-106-Ser mutation. White markers indicate biotypes and accessions with the Pro-106-Ser mutation, and black markers indicate those without the Pro-106-Ser mutation. Glyphosate resistance categories based on 80% survival at 0×(susceptible, S), 1×(R1), 8×(R2), 20×(R3), and 40×(R4). See Supplemental Table 1 for GPS locations for each biotype or accession. Satellite imagery © 2018 Google and map data provided by: Google, Image Landsat/Copernicus, and Image NOAA. The satellite images are used under fair use as noted at: https://www.google.com/permissions/geoguidelines/ and https://www.google.com/permissions/geoguidelines/attr-guide/. Outline of United States and Canada was retrieved from *Wikimedia Commons, the free media repository* located at https://commons.wikimedia.org/w/index.php?title=File:BlankMap-USA-states-Canada-provinces.svg&oldid=378653031 and licensed under the Creative Commons Attribution-Share Alike 2.5 Generic license (https://creativecommons.org/licenses/by-sa/2.5/deed.en). Composite figure was generated and adapted by Z.T. Beres.

The broad geographic distribution of the point mutation across northcentral Ohio, southern Ontario, and southern Iowa (Fig. 2), suggests multiple independent origins. Although seed dispersal could contribute to shared occurrences in southern Ontario and northcentral Ohio, the closest Iowa biotype to either an Ohio biotype or a Canadian accession was at least 700 km away (Fig. 2), and Dauer *et al*.^36,37^ estimated that heavily infested fields may potentially disperse horseweed seeds ~1–5 km per year. For non-target site glyphosate resistance, Okada *et al*.^38^ detected multiple origins in horseweed populations in California, where resistance was first reported in 2006^1^. For reference, glyphosate resistance was first reported in Ohio, Iowa, and Canada in 2002, 2011, and 2010, respectively^1^. Seed collections for the current study were carried out in 2015 (Ohio, Iowa) and 2011 and 2012 (Canada).

We suggest that the recent discovery of the p106 mutation conferring glyphosate resistance in horseweed can be attributed to previous methodology, in addition to the possibility that new occurrences of the p106 mutation are continuing to evolve due to heavy use of glyphosate. As noted above, most studies investigating the mechanisms of glyphosate resistance in horseweed did not characterize different levels of resistance (often solely using 1x or 2x as a discriminating dose) and used extremely small sample sizes for comparisons (1–2 susceptible populations and 1–2 resistant populations). While Page *et al*.^23^ did not characterize the levels of glyphosate resistance in their study, they did sequence a sufficiently large sample (N = 28 glyphosate-resistant populations), which may have improved the likelihood of detecting the point mutation. Studies that use small sample size comparisons and only low-level resistant (R1 or R2) biotypes might not be expected to detect a point mutation, given that the point mutation was associated with the extremely resistant (R3 and R4) biotypes in the present study (Table 2).

As shown in Table 1, all of the known cases of target-site mutations for glyphosate resistance in various weed species occur in the conserved region of *EPSPS* at p106, p102, and, in one case p103. Further research and literature reviews on target-site mediated glyphosate resistance would benefit from clear and consistent numbering of amino acid positions in *EPSPS*. In general, references to the p106, p102, and p103 mutations have become standard practice in research on herbicide resistance mechanisms, but this is not always the case. For example, some authors have referred to the p106 mutation as p182 based on alignment with *Arabidopsis thaliana*, GenBank:CAA29828.1^39^. Figure 1 shows a portion of the highly conserved region for several species with a reported Pro-106-Ser mutation, but based on GenBank accession numbers accompanying each publication, a proline does not occur at p106 in any of these species, including *Eleusine indica*.

In order to minimize confusion about convergent evolution of this point mutation in scientific publications, we propose adhering to the standard amino acid numbering system established by Baerson *et al*.^6^, which aligns the conserved region with microbial sequences, while also acknowledging the true position of a given substitution based upon species-specific sequences (Fig. 1). For example, in the present study, we refer to the proline to serine mutation occurring at p106, but we note in the Methods that this is actually position 185 based on the GenBank accession. Both numbering systems are needed to describe the position of this resistance mechanism and allow comparisons of conserved amino acid sequences across species. The ubiquity of the Pro-106-Ser mutation for glyphosate resistance, in particular, represents a classic example of convergent evolution, and a weak link in the long-term durability of glyphosate sensitivity within weed populations.

## Materials and Methods

### Study species

*Conyza canadensis* L. Cronq., a native North American species, has spread worldwide and is common in field margins, abandoned fields, roadsides, industrial areas, and other disturbed sites, in addition to row crops, orchards, vineyards, and other perennial crops^21,36,40^. Rosettes bolt to produce a ~1–2 m tall, multi-branched flowering stem^40,41^. Individual plants can produce >200,000 tiny, wind-dispersed seeds that exhibit no dormancy and are relatively short-lived in soil seed banks^40,42^. The seeds can disperse >500 km via the upper atmosphere^43^, but only ~1% of seeds disperse >100 m from their maternal plants^36^. Nonetheless, seeds from large, heavily infested fields could potentially disperse ~1–5 km per year^36,37^.

### Seed collections

This study used a subset of samples that were characterized for glyphosate resistance in two previous studies (see details in Beres *et al*.^32^; Page *et al*.^23^). Soybean-producing counties were selected for sampling in northcentral Ohio and southern Iowa, with a minimum of 1.6 km between collection sites in both states. Briefly, we collected seeds from one mature plant in each population during fall 2015 (Fig. 2; Supplemental Table 1). Because horseweed is highly selfing^14,44^, seeds from the same plant are assumed to be full siblings, and we refer to each maternal seed family as a “biotype.” We also used seeds from 10 Canadian populations: 5 glyphosate resistant populations which had the p106 mutation for serine, and 5 susceptible populations which did not have the mutation^23^ (Fig. 2). Canadian seeds were collected in 2011 and 2012 (see Page *et al*.^23^). We refer to these samples as accessions, rather than biotypes, because they represent bulked samples from populations that may or may not have a mixture of susceptible and resistant plants.

### Screening for glyphosate resistance

Methods for glyphosate resistance screening for biotypes originating from Ohio and Iowa are described in Beres *et al*.^32^. Canadian accessions had been scored as either susceptible or resistant using a different method by Page *et al*.^23^, so we screened them again to obtain resistance rankings that would be comparable to those reported for Ohio or Iowa. For biotypes from Ohio and Iowa, we used a randomized complete block design with five concentrations of glyphosate, three trays per biotype at each glyphosate concentration, and 6 plants per tray. For the Canadian accessions, we used two trays per accession at each of five glyphosate concentration and 4 plants per tray, except for the control (0x) tray, which had 2 plants per accession. As a method of quality control and reproducibility, we included three previously screened biotypes from Ohio while screening the Canadian accessions.

Seeds from each biotype or accession were germinated in trays (12 cm ×24 cm) with potting soil (Fafard 3B; www.fafard.com) mixed with a slow-release fertilizer (Osmocote, 14-14-14; www.osmocotegarden.com) to minimize nutrient deficiencies, based on manufacturer’s recommendations for annuals (~360 grams/2.8 ft^3^). Tray positions were randomized weekly to minimize environmental variation in the greenhouse. The greenhouse was maintained at 25/18 °C day/night with a 14-hour photoperiod, and plants were watered as needed. Seedlings were thinned 7 days after planting to leave 4 (2 for control) uniformly-sized and spaced plants within each tray.

All spray treatments included ammonium sulfate solution (N-Pak® AMS Liquid, 407 g L^−1^; Winfield Solutions, LLC; St. Paul, Minnesota) and non-ionic surfactant (Preference®; Winfield Solutions, LLC) at 5% and 0.5% (v/v), respectively. When rosettes were 4–6 cm in diameter, the plants were treated with one of five glyphosate doses: 0x(ammonium sulfate solution and surfactant only), 1x[840 g ae ha^−1^; manufacturer’s recommended application rate, which equates to 0.6725% glyphosate (v/v); AquaMaster®, 648 g L^−1^, Monsanto Co.; St. Louis, Misouri], 8x, 20x, or 40x. This rosette size range at application was similar to previous studies of GR horseweed^21,32,45^. Treatments were applied using a pneumatic track sprayer equipped with an even, flat-spray tip (Teejet 8001EVS; Spraying Systems Co.; Carol Stream, Illinois) calibrated to apply 140 L ha^−1^ of spray solution at 3.5 km hr^−1^. Plants were returned to the greenhouse and maintained for 3 weeks as previously described.

At 21 days after treatment, individual plants were visually assessed for leaf damage on a scale from 0 (no damage) to 10 (dead), with 9 being ~90% dead tissue. Proportion survival was calculated for each biotype/accession at each dosage. The proportion surviving at each dosage was used to characterize each biotype or accession into resistance categories. Biotypes with less than 80% survival at 1x were classified as “Susceptible.” Resistant biotypes with 80% survival at 1x but less than 80% survival at 8x were classified as “R1.” Likewise, biotypes with >80% survival at 8x were classified as “R2,”>80% survival at 20x as “R3,” and >80% survival at 40x as “R4.”

### DNA extraction

Horseweed seeds from the same biotypes or accessions described above were germinated during spring 2018 in 3” round pots filled with moistened Fafard no. 2 soil. Plants were thinned one week after planting to leave one seedling per pot (1 plant per biotype/accession x 54 biotypes/accessions = 54 plants total). The greenhouse was maintained at 18–21/23–26 °C (night/day), and supplemental lights (400-W metal halide) were used for 14-hour days.

After ~3 weeks in the greenhouse, 2 leaves from each plant were harvested (~200 μg of leaf tissue), snap frozen in 2-mL tubes in liquid nitrogen, and stored at −80 °C. One leaf was used to collect DNA while the second leaf remained in storage as a back-up sample. Tissue samples were ground in 2-mL tubes by shaking with two 5-mm glass beads at 30-Hz for 1 minute with a Qiagen Tissue Lyser in Tissue Lyser plates that had been pre-chilled to −80 °C. Genomic DNA was then extracted using a DNeasy Plant Mini Kit from Qiagen (#69104), quantified with a NanoDrop™ spectrophotometer, and stored at −20 °C until use.

### PCR and sequencing

Based on findings of Page *et al*.^23^, we focused on sequencing DNA from exon 2 in *EPSPS*2. PCR was used to amplify a 986-bp region (GenBank Accession: AY545667.1) around and including the expected single nucleotide polymorphism (SNP) at p106 (denoted as p185 based on the GenBank accession; see Results & Discussion) of the *EPSPS*2 gene. The designed primers ensured coverage of the p102 and p106 sites because these are the most common positions for a point mutation conferring glyphosate resistance. The forward primer had a sequence of ggactactgttgtagacaacttg, and the reverse primer was gtgggcagtttgtaccgaga. The PCR reactions were run under standard conditions with Taq from New England Biolabs (#M0267L). The thermal cycler program started with an initial denaturation at 95 °C for 2 min, followed by 35 cycles of 95 °C for 20 sec, 58 °C for 30 sec, and 68 °C for 1 min. After cycling was complete a final extension at 72 °C for 5 min was performed to end the PCR reaction. PCR products were held at −20 °C until they were sequenced.

Reactions that produced a product of 986-bp were cleaned with a kit from Qiagen (#28104) to remove excess reagents and primers. The OSU Genomics Shared Resource at The Ohio State University performed sequencing of the clean PCR products. The sequencing primer had a sequence of gaatcctcctactcatataattgtg. Sequence data was analyzed using SnapGene viewer (version 3.0.1) to identify any point mutations, especially a proline to serine substitution. A single nucleotide polymorphism (SNP) at position 106 of *Conyza canadensis EPSPS*2 cDNA was scored as either “C” encoding proline or “T” encoding serine at amino acid position 106 (Fig. 1; Table 2). No other SNPs were identified in any of our samples.

## Supplementary information

Supplementary Information.
