# Supplementary material for: Target-site EPSPS Pro-106-Ser mutation in Conyza canadensis biotypes with extreme resistance to glyphosate in Ohio and Iowa, USA
Source: Sci Rep. 2020 May 5;10:7577. doi: 10.1038/s41598-020-64458-7 (PMC7200745; doi:10.1038/s41598-020-64458-7)
Supplement: Supplementary file 1 — Supplementary Information. [file 41598_2020_64458_MOESM1_ESM.docx]

**Target-site *EPSPS* Pro-106-Ser mutation in *Conyza canadensis* biotypes with extreme resistance to glyphosate in Ohio and Iowa, USA**

Zachery T. Beres, Laura A. Giese, David M. Mackey, Micheal D. K. Owen, Eric R. Page, and Allison A. Snow

Supplemental Table 1. Locations of sampled biotypes and glyphosate resistance category for biotypes and accessions used. “S” and “N” in front of sample IDs designate samples collected from “Soybean fields” or “Non-soybean sites,” respectively. Resistance categories based on 80% survival at 0x only (S = susceptible), and up to 1x (R1; equivalent to 840 g ae ha^-1^), 8x (R2), 20x (R3), and 40x (R4).

| Region | Sample ID | Resistance Category | Latitude | Longitude |
| --- | --- | --- | --- | --- |
| Ohio | N64 | S | 41.04759 | -81.68773 |
|  | N28 | S | 40.84761 | -81.82333 |
|  | N50 | S | 40.68304 | -82.30898 |
|  | N53 | S | 40.63685 | -82.31474 |
|  | N58 | S | 40.5906 | -82.38384 |
|  | N66 | S | 41.10828 | -81.80928 |
|  | N90 | S | 40.4623 | -82.12644 |
|  | N42 | R1 | 40.5976 | -82.1123 |
|  | N83 | R1 | 40.46176 | -81.96639 |
|  | N52 | R2 | 40.63522 | -82.26362 |
|  | N89 | R3 | 40.50764 | -82.05477 |
|  | N11 | R4 | 41.16946 | -82.27295 |
|  | S15 | R4 | 41.02955 | -82.3879 |
|  | S18 | R4 | 40.95922 | -82.24953 |
|  | S23 | R4 | 40.91577 | -82.02224 |
|  | N26 | R4 | 40.91626 | -81.84684 |
|  | S3 | R4 | 41.36073 | -82.297113 |
|  | S38 | R4 | 40.70835 | -82.08995 |
|  | S60 | R4 | 40.59641 | -82.4358 |
|  | N65 | R4 | 41.04932 | -81.77804 |
|  | S74 | R4 | 41.11687 | -82.11356 |
|  | N76 | R4 | 40.63496 | -81.68366 |
|  | S78 | R4 | 40.48928 | -81.67429 |
|  | N9 | R4 | 41.26045 | -82.15756 |
| Iowa | N17 | S | 40.79477 | -92.09364 |
|  | N2 | S | 41.29313 | -94.45269 |
|  | N9 | S | 41.94282 | -93.85949 |
|  | N12 | R1 | 40.83422 | -92.71984 |
|  | N19 | R1 | 41.68511 | -91.91067 |
|  | S16 | R1 | 40.85336 | -92.10659 |
|  | S4 | R1 | 41.74592 | -92.66809 |
|  | S8 | R1 | 41.96507 | -93.92205 |
|  | S37 | R2 | 41.95733 | -94.20398 |
|  | N10 | R3 | 40.72873 | -92.78172 |
|  | S12 | R3 | 40.79292 | -92.62881 |
|  | S9 | R3 | 41.28706 | -93.06057 |
|  | N28 | R4 | 40.94598 | -92.63812 |
|  | S11 | R4 | 40.76117 | -93.49468 |
|  | S14 | R4 | 40.74769 | -92.35134 |
|  | S15 | R4 | 40.84683 | -92.09708 |
|  | S25 | R4 | 40.85530 | -93.47446 |
|  | S27 | R4 | 40.85628 | -93.69547 |
|  | S41 | R4 | 40.97446 | -91.70406 |
|  | S45 | R4 | 41.19199 | -91.52598 |
| Canada | 59 | S | 42.83088 | -81.87118 |
|  | 67 | S | 42.9175 | -81.60435 |
|  | 75 | S | 43.00421 | -79.36771 |
|  | 79 | S | 43.59633 | -81.47631 |
|  | 80 | R1 | 43.60353 | -81.47039 |
|  | 20 | R3 | 42.032082 | -82.90276 |
|  | 10 | R4 | 42.08816 | -83.09339 |
|  | 13 | R4 | 42.16001 | -82.85863 |
|  | 15 | R4 | 42.55968 | -81.80225 |
|  | 27 | R4 | 42.22336 | -82.99233 |
